# Queensland Alcohol-related violence and Night Time Economy Monitoring project (QUANTEM): a study protocol

**DOI:** 10.1186/s12889-017-4811-9

**Published:** 2017-10-05

**Authors:** Peter G. Miller, Jason Ferris, Kerri Coomber, Renee Zahnow, Nicholas Carah, Heng Jiang, Kypros Kypri, Tanya Chikritzhs, Alan Clough, Michael Livingston, Dominique de Andrade, Robin Room, Sarah Callinan, Ashlee Curtis, Richelle Mayshak, Nicolas Droste, Belinda Lloyd, Sharon Matthews, Nicholas Taylor, Meredythe Crane, Michael Thorn, Jake Najman

**Affiliations:** 1 School of Psychology, Deakin University, Waterfront Campus, Geelong, VIC 3220 Australia; 2 School of Communication and Arts, University of Queensland, Brisbane, QLD Australia; 3 Institute for Social Science Research, University of Queensland, Brisbane, QLD Australia; 4 Centre for Alcohol Policy Research, LaTrobe University, Melbourne, VIC Australia; 5 School of Medicine and Public Health, University of Newcastle, Newcastle, NSW Australia; 6 National Drug Research Institute, Curtin University of Technology, Perth, WA Australia; 7 College of Public Health, Medical & Vet Sciences, James Cook University, Cairns, QLD Australia; 8 School of Psychology and Counselling, Queensland University of Technology, Brisbane, QLD Australia; 9 Turning Point Alcohol and Drug Centre and Monash University, Melbourne, VIC Australia; 10 Foundation for Alcohol Research and Education, Canberra, ACT Australia; 11 Queensland Alcohol and Drug Research and Education Centre, School of Public Health, University of Queensland, Brisbane, QLD Australia

**Keywords:** Alcohol, Policy, Evaluation, Protocol, Australia

## Abstract

**Background:**

Alcohol-related harm is a substantial burden on the community in Australia and internationally, particularly harm related to risky drinking practices of young people in the night-time economy. This protocol paper describes a study that will report on the changes in a wide range of health and justice outcome measures associated with major policy changes in the state of Queensland, Australia. A key element includes trading hours restrictions for licensed premises to 2 am for the state and 3 am in Safe Night Precincts (SNPs). Other measures introduced include drinks restrictions after midnight, increased patron banning measures for repeat offenders, mandatory ID scanning of patrons in late-night venues, and education campaigns.

**Methods:**

The primary aim of the study is to evaluate change in the levels of harm due to these policy changes using administrative data (e.g., police, hospital, ambulance, and court data). Other study elements will investigate the impact of the Policy by measuring foot traffic volume in SNPs, using ID scanner data to quantify the volume of people entering venues and measure the effectiveness of banning notices, using patron interviews to quantify the levels of pre-drinking, intoxication and illicit drug use within night-time economy districts, and to explore the impacts of the Policy on business and live music, and costs to the community.

**Discussion:**

The information gathered through this project aims to evaluate the effectiveness of the Policy and to draw on these findings to inform future prevention and enforcement approaches by policy makers, police, and venue staff.

**Electronic supplementary material:**

The online version of this article (10.1186/s12889-017-4811-9) contains supplementary material, which is available to authorized users.

## Background

Governments have implemented a variety of legislative amendments, regulations, and programs that aim to address risky drinking practices by restricting access to alcohol and/or deter offensive behaviour. Reducing trading hours, restrictions on outlet (venue) density, education campaigns, and price increases are some of the strategies employed to reduce alcohol-related harms (e.g., [1]). While there is strong evidence that increases in trading hours increase the rate of alcohol-related assaults and injuries [2–4], the impacts of other strategies such as education campaigns are less clear. This is largely owing to the fact that, typically, alcohol policies comprise multiple strategies, introduced simultaneously. For example, the New South Wales Liquor Amendment Act 2014 included earlier cessation of alcohol sales, lockout (one-way door) conditions, risk-based licensing fees, a ban on takeaway sales after 10 pm, and extension of banning orders to prevent troublesome patrons entering key entertainment areas. The recently implemented Queensland Government’s Tackling Alcohol-Fuelled Violence Policy 2016, draws heavily on this approach.

The tendency towards multi-pronged approaches for addressing alcohol-related violence makes policy evaluation of a single intervention or strategy extremely difficult. Disentangling the effects of individual components is particularly challenging, as is identifying aspects of the policy that have little discernible impact when considered in isolation [1]. We have framed this evaluation in terms of ‘complex interventions’ as per the United Kingdom Medical Research Council guidance to weigh up the available evidence in the light of these methodological and practical constraints, ensuring there is consideration of all elements of the intervention (through process and outcomes), as well as monitoring the intervention through different stages to map changes over time [5].

### Queensland government alcohol policy

**Fig. 1:**
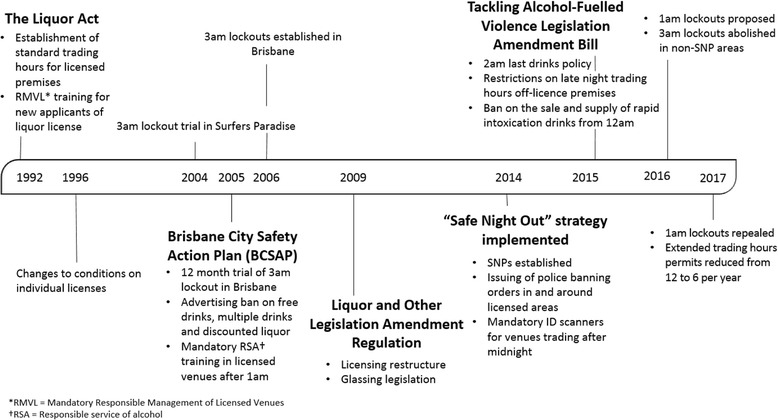
Timeline of liquor licencing responses in Queensland since 1996

In 2016, the Queensland Government responded to the community’s concerns around alcohol-fuelled violence and other harm by implementing a broad-based multi-faceted policy. While this is not the first multi-faceted policy concerning alcohol problems that a Queensland Government has introduced (see Fig. 1), this is the first time earlier last-drinks legislation has been implemented at a state wide level, rather than within a few key entertainment districts.

The components of the Queensland Government’s Tackling Alcohol-Fuelled Violence Policy are outlined in Table 1. Most components of the policy are consistent across the state, with some applicable only to designated Safe Night Precincts (SNPs). There are currently 15 SNPs (key entertainment areas that are managed by local boards) across Queensland. The Policy has three broad aims [6]:

1. To achieve cultural change around drinking behaviour which includes more responsible drinking practices (in particular drinking behaviour outside of the home and in designated SNPs in Queensland),
2. A safer night time environment, in particular in entertainment precincts, and
3. A regulatory framework that balances the interests of the liquor industry with a reduction in alcohol-fuelled violence.

**Table 1:** Tackling Alcohol-fuelled Violence Legislation Policy 2016

Elements of the Policy commenced prior to 1 July 2016
• existing applications to extend hours for the sale of takeaway liquor beyond 10 pm to be voided, with no new applications to be accepted, though existing approvals to sell takeaway liquor will remain – from 4 March 2016 (the date of assent of the Tackling Alcohol-Fuelled Violence Legislation Amendment Bill 2015); • the continuation of the 15 Safe Night Precincts, including rest and recovery services (also called Safe Spaces), around the State; • the continuation of the local board model for management of Safe Night Precincts, including the grant funding pool for alcohol-related violence initiatives in Safe Night Precincts; • targeted drug and alcohol assessment and referral programs for alcohol- and drug-related offences; • the discontinuation of the Sober Safe Centre initiative, and instead, continuing the trial of paramedics in watch-houses; • the continuation of compulsory education in schools about alcohol- and drug-related violence; • the continuation of social marketing about alcohol- and drug-related violence; • the continuation of implementation oversight mechanisms.
Elements of the Policy commenced on 1 July 2016
• regular service hours for alcohol in licensed venues across Queensland ends at 2 am, except for premises located within a Safe Night Precinct approved for 3 am trading; • A ban on the sale of rapid intoxication, high alcohol-content drinks after midnight; • 3 am lockouts removed.
Elements of the Policy commencing after 1 July 2016
• introduction of mandatory operation of networked ID scanners in late-night trading licensed premises located in Safe Night Precincts (to commence 1 July 2017); • extension of the powers of courts to impose banning orders on persons convicted of drug offences in Safe Night Precincts. These changes will be subject to consultation with stakeholders and would require legislative changes. • extended trading permits restricted to 6 per venue per calendar year (previously 12 per year as per existing policy).
Elements of the Policy repealed prior to introduction
• introduction of 1 am lockout in prescribed 3 am Safe Night Precincts – repealed (was to commence on 1 February 2017)

### The current study

The aim of the project outlined in this protocol is to assess the impact of the Policy on patterns of consumption and alcohol-related harms in Queensland, while also identifying unintended consequences of the Policy on the night-time economy. The approach to this policy evaluation is unique in terms of the breadth of data sources available, and comprehensiveness of perspectives considered.

Drawing on administrative data (e.g., police, ambulance, emergency, courts, and hospital admissions and emergency department attendances), we will model key indicators of alcohol-related harms pre- and post- introduction of the Policy. The indicators selected for this evaluation are well-established in the international literature as reliable and valid [7]. Our evaluation also considers novel sources of information including foot traffic counts, patron and key stakeholder interviews, and venue observations which will allow for important additional insight into questions regarding causality and mechanisms of change (e.g., was a reduction in assaults driven by a reduction in the number of people in a SNP or a reduction in levels of intoxication), as well as third party administrative data including ID scanner data.

## Methods

### Study design

The study employs a mixed-methods design involving multiple data collection components and analysis of administrative data sources, designed with the strengths of complex interventions framework in mind [7–9]. The core evidence of the impact of the Policy interventions will be derived from changes in key outcomes such as police, emergency department (ED), hospital admissions, and ambulance data. To provide greater insight and context into how these impacts are achieved and what other (sometimes unintended) consequences arise we will collect and analyse a range of supplementary information. There are seven key components to the study methodology:

1. Administrative data analysis (harms and business-related);
2. Patron interviews (including follow-up surveys);
3. Key stakeholders interview;
4. Structured venue observations;
5. Precinct streetscape and business mapping;
6. Foot traffic counting;
7. Economic evaluation.

### Setting

This study will be undertaken state-wide across Queensland, Australia, with a particular focus on designated night-time entertainment districts (or safe night precincts: SNPs). SNPs were established in Queensland in 2014 under the Government’s Safe Night Out Strategy. The 15 SNPs in Queensland represent key entertainment areas with high densities of licensed venues and have special conditions in place around the trading of alcohol (for maps of the precincts see [10] or Additional file 1). This study focuses on five purposively selected SNPs: Fortitude Valley (an inner city suburb of Brisbane); Cairns, Townsville, Surfers Paradise (Gold Coast), and Toowoomba. The range of sites, across the state, have been selected to be as representative of different types of nightlife as possible. Fortitude Valley and Surfers Paradise are the largest nightlife precincts in the state, Cairns is a tourist destination in the far north of the state, Toowoomba has few tourists and is an inland community. West End (another inner city suburb of Brisbane) is also included as a specific non-SNP comparison site.

To assess state-wide impacts of the Policy, a comparison of Queensland administrative data (state wide and for specific precincts) to other Australian states will be undertaken. Interstate comparison sites have been primarily selected based on the size of their night-time economies, and their resident population aged 18–30 (see Table 2). Table 3 provides a summary of study sites and project elements.

**Table 2:** Administrative data research sites and comparison sites

Queensland sites	Population	N total licensed venues	N late night venues	Comparison site/s	Population	N licensed venues	N late night venues
Brisbane (Fortitude Valley)	1,162,186	186	40	Perth (CBD + Northbridge)	21,092	294^d^	28
				West End (Qld)	1,162,186	64^b^	0
Cairns	160,285	232	50	St Kilda (Vic)	107,127	178^e^	34^e^
				Noosa Heads/Noosaville (Qld)	56,151	103	7
Townsville	193,946	97	43	Adelaide (CBD)	23,169	435	34^f^
				Greater Newcastle	308,308	146^g^	32^g^
Gold Coast (Surfers Paradise)	555,608	166	38	Melbourne (Chapel St)	107,941	137^e^	54^e^
Toowoomba	163,232	126	30	Geelong (Vic)^h^	229,420	257^e^	14^e^
				Greater Newcastle	308,308	146^g^	32^g^

License counts excludes packaged, limited, producer, and wholesaler licenses; indicative numbers only

^a^Local Government Area Population as at 2015

^b^As at 29 August 2016; Queensland Office of Liquor Gaming and Regulation (OLGR)

^c^As at 2015; sourced from Queensland Government Fortitude Valley and Surfers Paradise Drink Safe Precinct trial reports and OLGR annual report

^d^As at 29 August 2016; Department of Racing, Gaming, and Liquor

^e^As at 29 August 2016; Victorian Commission for Gambling and Liquor Regulation

^f^As at 2015; industry figures. Subject to data availability. Greater Newcastle will be used if data is not released

^g^As at 2015; New South Wales Liquor and Gaming

^h^Geelong will be used as an alternate if Newcastle is used for Townsville

**Table 3:** Study elements by site

Site/Element	Administrative data	Patron interviews	Follow-up survey	Key informant interviews	Venue observations	Precinct streetscape, business mapping	Economic evaluation	Foot traffic	ID scanner data	School AOD program audit
Queensland wide	✓						✓		✓	✓
Fortitude Valley	✓	✓	✓	✓	✓	✓		✓		
Cairns	✓	✓		✓		✓		✓		
Gold Coast	✓	✓		✓		✓				
West End	✓	✓	✓	✓	✓	✓				
Townsville	✓					✓				
Toowoomba	✓					✓				
Interstate comparison sites										
Victoria wide	✓									
Perth, WA^b^ (CBD^a^ + Northbridge)	✓									
St Kilda (Vic)	✓									
Adelaide, SA^c^(CBD)	✓									
Melbourne, Vic^d^ (Chapel St)	✓									
Geelong (Vic)	✓									
Greater Newcastle (NSW^e^)	✓									

*AOD* alcohol and other drugs;

^a^Central business district

^b^Western Australia

^c^South Australia

^d^Victoria

^e^New South Wales

### Procedures and measures

#### Administrative data – Alcohol-related harms

This evaluation draws on a range of administrative and archival data sources to provide a comprehensive assessment of the impact of the Policy. All administrative data will be used to assess trends in alcohol-related harms before and after the Policy introduction. Patterns of harms during high-alcohol hours (HAH) will be examined for much of this data, given this is the time period the legislation mostly focusses on and when most harms are identified [2, 11]. HAH for police and ambulance data are defined as Friday and Saturday nights, 8 pm to 6 am [12], whereas HAH for emergency departments is defined as Friday and Saturday nights, midnight to 5 am [13]. Research demonstrates that the use of such surrogate measures are reliable for assessing trends over time [12, 14–16]. All administrative data will be de-identified.

##### Police assaults data

Police assaults data are provided by the Queensland Police Service (QPS) for assault offences occurring from January 2000 onwards. QPS data are extracted from the Queensland Police Records and Information Management Exchange (QPRIME) database. QPRIME data contain unit level records of crime incidents, core fields include: type of offence; age and sex of perpetrator and victim(s); types of location (i.e., licensed outlet, house/unit, street etc.); geographic location (i.e., postcode); domestic violence indicator; and date and time of day of offence.

QPRIME data contains nine assault classifications. We will distinguish between common and serious assaults. Common assaults include: assaults, common and minor assaults (not elsewhere classified). Serious assaults include: assault occasioning bodily harm; assault, aggravated (non-sexual); assault, police; assault, serious (other); grievous bodily harm; wounding. The key outcome variable will be the number of assault offences during HAH.

##### Emergency department injury attendance data

De-identified ED unit records for Queensland hospitals will be used to assess the impact of the Policy on late-night injuries. The ED data comes from the Non-Admitted Patient Emergency Department Care (NAPEDC) database. This data captures a broad spectrum of alcohol-related injuries (e.g., minor fractures from falls and assaults), many of which are unlikely to be recorded in police data [17, 18], and are often not serious enough to result in admission to a hospital ward, but occur relatively frequently among high-risk population groups [19]. The ED data will include 28 Queensland hospitals from January 2005 onwards. Core fields in the ED data include: primary diagnosis; patient age and gender; time/date of presentation; postcode; and a location description where injury occurred (e.g., at home, on street). ED data do not contain a reliable indicator of patient intoxication or alcohol involvement in the incident preceding presentation nor do they include external cause codes; these omissions preclude differentiating injuries due to violence.

##### Hospital admissions data

Hospital admissions data will also allow examination of trends in serious injuries resulting in hospitalisation, thus providing a comprehensive picture of the burden of acute alcohol-related harm on the health system. Hospital records are available from January 2005 onwards and will be obtained through the Queensland Hospital Admitted Patient Data Collection (QHAPDC). Based on previous research methodology [20], hospital records related to alcohol are extracted based on the ICD-10-AM codes [21] of the principal diagnosis associated with the hospitalisation as well as additional details related to the individual and the hospitalisation: patient’s date of birth, gender, postcode; as well as hospital admission and separation date and time; date of incident; and length of stay.

##### Ambulance data

Ambulance data are a valuable adjunct to police and ED data for monitoring different types of alcohol-related harm that do not result in either ED presentation or a police offence being recorded [22–24]. The data are sourced from the Queensland Ambulance Electronic Ambulance Report Form (EARF) and Queensland Ambulance Case Information Reporting (QACIR) databases. The Queensland Ambulance Service data contain unit level records of all ambulance attendances in Queensland; core fields include: primary reason for attendance; patient age and gender; time/date of attendance; postcode; address; and if the case was taken to hospital or treated on site. As there is no reliable indicator of alcohol involvement in the dataset, the data extraction will involve keyword searches and use of relevant diagnostic codes for cases which occur during HAH, which has previously been found a reliable proxy [25].

##### Courts data

Data pertaining to prosecutions of licensees for breach of liquor licence conditions or service of intoxicated patrons has been obtained from the Magistrates Court to assess the use of a legislative amendment allowing for the breathalysing of intoxicated or disorderly patrons for the purposes of gathering evidence of venues serving unduly intoxicated patrons. The use of police and court invoked patron bans is another aspect of the policy that will be investigated using court and police data. Outcomes for offenders diverted to the Drug and Alcohol Assessment Referral course (commenced July 2016) compared to those who do not participate in this program will also be evaluated.

#### Administrative data – Business data

In addition to examining trends in alcohol-related harms, this project use business data to identify the economic and cultural impacts of the Policy. Key sources of business data are outlined below.

##### Office of Liquor and Gaming Regulation (OLGR)

Data from the Office of Liquor and Gaming Regulation has been used to establish the spatial distribution of licensed venues in Queensland and the density of venues in night time entertainment areas between 1 January 2010 and 1 July 2018. A list of venues licensed to sell liquor in Queensland, including address, license type and licensed hours of operation, has been sourced from OLGR. Additionally, OLGR will provide data on applications for extended trading permits. Core fields include: venue name, license number, date and time of extended trade. This data is required to assess the number of hours of liquor sales on a weekly basis.

##### Australian business survey (ABS) and Australian business register (ABR)

Publically available data routinely collected by the Australian Bureau of Statistics on the number of businesses with no employees, 1–4 employees, 5–19 employees, 20–199 employees, over 200 employees, and total employees by industry in Queensland has been collected [26]. For each of the six study sites (Fortitude Valley; Surfers Paradise; Townsville; Toowoomba; Cairns; and West End), this data will be joined with data from the Australian Business Registry (ABR). The ABR data contains the physical address, industry type and date of registration for all registered businesses. The database is dynamic and regularly updated. Data will represent snapshots at 6 monthly intervals to examine changes in the number of registered businesses at each site at each time point. When coupled with the ABS employee count data (published annually), this data can be used to assess trends in business entry and exit and employment in the six study sites within the liquor industry compared to other industry types.

#### Precinct streetscape and business mapping

This component of the study will involve members of the research team systematically documenting the nature of all business occurring in the declared night time entertainment precincts over the course of a day and night. ‘Walkthroughs’ will be conducted at each of the six primary sites at six month intervals to document:

- Change in venue size, diversity and density over time.
- Changes in the number and range of venues open throughout the night up until legislated closing times.
- Changes in actual venue opening hours, both opening and closing earlier, relative to licences.
- Number and type of businesses closing down and opening up.

The walkthroughs will occur on Saturday evening at set intervals: 10 pm, 12 am, 2 am, and 4 am. Data collected from the walkthroughs will be used to develop precinct maps for Fortitude Valley, West End, Surfers Paradise, Toowoomba, Cairns, and Townsville that plot each business in the precinct (including venues, restaurant, food outlet, bar or performance spaces) and record the opening hours, entertainment, and food and beverage offerings for each venue. Over a two-year period, four maps will be created to enable an analysis of changes in venue mix and density, diversity of entertainment options and cultural performances, and mix of food and beverages over time.

#### Patron interviews and surveys

##### Patron interviews

Street-intercept, patron interviews will be conducted over a 2-year period. Interviews began 4 weeks prior to the legislation being introduced. While not ideal in terms of demonstrating impact pre and post, it will supply some insight into trends prior to the implementation of the policy, with appropriate caveats. The key role of the patron interview is to document patrons’ experiences of violence in the night time environment, evaluate patron responses to the legislative changes, and describe any self-reported behaviour change (e.g., pre-drinking, time at which patrons go out to licensed premises). The interview strategy is informed by previous projects (e.g., [11, 27]). Researchers will work in groups of six in public thoroughfares in key entertainment areas with every third patron on the street invited to participate in an interview. Once participants provide verbal consent to be interviewed, they are given a business card that will have a web address and contact details of the study investigators and ethics committee, which they may use if they wish to know more about the study or be informed of study findings. Data collection will occur on Saturday nights between 10 pm and 5 am (exact times within this frame may vary dependent on day and location), with an interview length of 5–15 min.

Every patron interviewed is breathalysed and blood alcohol concentration (BAC) levels recorded within the interview data, tracking levels of intoxication throughout the night [28]. Every fifth person interviewed will also be asked to do a drug saliva test.

Patron interviews will be conducted in Brisbane (Fortitude Valley and West End), Surfers Paradise, and Cairns. Interviews will be monthly in Fortitude Valley and Cairns, and every second month in West End and Surfers Paradise. Monthly sessions were chosen to allow mapping of trends over time and seasonally, within budgetary constraints.

The patron interview has five components:

1. *Demographics:* gender; postcode; age; occupation.
2. *Current night out:* time of arrival at the night time precinct; hours spent drinking; perceived level of intoxication; feelings of safety; engagement in pre-loading, their use of energy drinks, their use of illicit substances.
3. *Experiences of violence/alcohol-related consequences (past 3 months):* experiences of physical, verbal or sexual aggression around licensed venues; the role of alcohol and drugs in these incidents; personal injury or accidents as a result of alcohol or drug use; engagement in offending such as property damage or drink driving; experiences of being ejected from a venue, refused service or refused entry.
4. *Changes in behaviour following the Policy and awareness of media campaign:* how has the Policy changed their drinking/partying behaviour; are they aware of any Government anti-violence campaigns.
5. *Intentions for the rest of the night:* their plans for getting home; their plans for the rest of the evening.

##### Follow-up patron survey

Patron interview participants in Fortitude Valley and West End will also be asked to do a follow-up, online survey that they can access from the next day (for up to 1 week) for a small reward [29]. The aim of the follow-up survey is to explore the participants’ activities for the remainder of their night out. Participants in Fortitude Valley and West End are asked to provide either an email address or mobile phone number to which the survey link can be sent. This follow-up method has been successfully trialled previously in Canada on 170 participants, with 68% (64% male, 75% female) of street survey participants completing the online survey [29]. Response rates for heavy drinkers was 53%. Online follow-up survey completers were similar to those who did not respond, although they generally pre-drank less.

The follow-up survey will take approximately 15–20 min and will include questions on: venues participants visited and experiences from the night before; alcohol-related incidents/consequences (injury, assault, and regretted behaviour); how much participants spent; the amount of alcohol consumed and substance use. A sample of 500 participants who complete the survey per site will be reimbursed $20 (gift card) for completing the survey. Power calculations indicate that this sample size will provide sufficient power to detect moderate to small effects in binary logistic regression models and random linear regression models (Odds Ratio: 1.4, R^2^ = 0.1 respectively) with a minimum power level of 0.8 and α error probability of 0.05. [30].

#### Key informant interviews

The study also involves key informant interviews with at least 50 selected individuals to inform the interpretation of the findings from administrative data analysis. These interviews will provide substantial insight into potential benefits and side-effects of the Policy which are not apparent from other data sources [2], and help to develop a comprehensive picture of impacts of the legislative changes.

The sampling frame includes five people per site from five key sectors of: government policy makers; service providers (e.g., night chaplaincy or rest and recovery services), hotel licensees (or hotels association); police; licensing personnel, relevant local council employees and health professionals (up to 25 per site). Only one person will be interviewed at a time and informed consent will be obtained. Interviews will normally be tape-recorded, but may also be an email or taped telephone interview. Stakeholders will be asked questions based on a series of prompts, rather than a strict set of questions, focussing on barriers to implementation of late night alcohol restrictions, perceptions of impact, recommendations for improvement and the identification of other relevant factors. Our previous research has demonstrated the valuable contribution of key informant interviews and their ability to provide policy-relevant insights into both the effectiveness of certain measures and how they can be implemented better [31, 32].

#### Structured venue observations

Observations will be conducted inside purposively selected venues at the Fortitude Valley and West End sites. The observations provide a source of information about nightlife culture and the type of entertainment provided, physical characteristics of venues, crowd density and compliance with liquor legislation. Coupled with the patron interviews, the venue observations provide context and aid in the interpretation of the administrative data analyses. Four rounds of observations will be conducted at each of the two sites over a 12-month period. Observations will be conducted by researchers in pairs. Each observer will conduct their observations independently and observe different parts of the venue. Therefore, each observation record will be treated as independent. The research includes the use of observation checklists, forms and technology developed from previous studies [33, 34]. Each pair of researchers will observe two venues per night, spending approximately 2.5 h in each venue. Observations will be as unobtrusive as possible. Researchers will be trained to covertly complete observation forms on an iPod touch screen, in line with previous studies [35, 36].

Key outcome variables include: the overall levels of intoxication observed; the number of highly intoxicated people who are subsequently served alcohol (three signs of intoxication); compliance to restrictions on alcohol service (e.g. no shots after 12 midnight); and compliance to mandatory ID scanning. Levels of alcohol consumption and observed illicit drug consumption will be recorded. We will also record the extent to which physical features of the venue comply with domains of the Crime Prevention Through Environmental Design (CPTED) framework, including: surveillance, access control, target hardening, image management, and activity support [37].

#### Foot-traffic counts

A limitation of previous evaluations of alcohol-related policy is the reliance on counts of alcohol-related incidents without any data regarding whether there were changes in the numbers of people attending the area. This is owing to the difficulties associated with estimating a population base to serve as the denominator in a calculation of incidence rates, although the data collected for this study can only identify trends over time, and cannot provide accurate population on-the-night estimates. To begin to address this limitation, this project will collect foot traffic data at two of the primary sites: Fortitude Valley and Cairns SNP. Although the use of a single counter means we are unable to accurately measure the total number of people in any nightlife area, we can document trends in a specific area over time, giving more insight than previous studies into any substantial changes in the number of people attending entertainment districts. This data provides a proxy for person density within each location.

People entering the night-time entertainment precincts in Fortitude Valley and Cairns are counted via the use of a wireless sensor [38]. This wireless sensor is placed within a shopfront in each precinct, and is calibrated and managed by Kepler Analytics. The data is processed by Kepler Analytics, and automatically visualised on a secure cloud based Dashboard, which the research team can access.

Data collection has been under way using this method since March 2016. The sensor captures mobile phone Wi-Fi signals, and records the number of people broadcasting a phone Wi-Fi signal within a particular area.

#### Economic evaluation

A cost-benefit analysis of the Policy will be conducted. The major estimated costs are the potential loss of revenue of affected licenced premises, the potential loss of income for the alcohol industry, and the potential loss of local government revenue. The major anticipated benefits are reduced violence, savings in health care costs, increased amenity for the neighbourhood, income gained by non-alcohol-related business, and the productivity gain due to reduced assaults and injuries. It should be recognised that a reduction in drinking, particularly for heavier drinkers, will also result in gains in chronic health status, reducing costs for health care and of lost productivity, but these will not be included in this analysis. Using administrative data described above, the cost-benefit analysis will proceed as follows:

1. Using pre- and post-intervention data (police assault, ED admissions, and ambulance data) to determine how changes in closing times and other measures in the Policy affected levels of alcohol-related social and health outcomes, as discussed in the administrative data analysis section [39],
2. Estimate consequences of earlier closing on local licenced venues (costs and benefits to business and community),
3. Estimate costs and benefits of the restriction for local government,
4. Estimate costs and benefits to late night drinkers and other private parties affected by late-night drinkers, for example reduced violence and injuries, and reduced health care costs; night time drinkers may travel to a non-restricted area for drinking.
5. Estimate benefits of impact on other industries and households.
6. Aggregate total costs and benefits to estimate net cost-benefit from a whole of society perspective.

#### Additional study elements

There are additional study elements being conducted which do not form a part of the core evaluation which will greatly assist in describing contextual elements around the intervention.

##### ID scanner data

De-identified, unit record data from major ID scanner suppliers (e.g., Scantek) will be analysed to better understand dynamic population flows in SNPs. ID scanning data provides information on the number of people entering a venue over the course of the night, the times at which they entered, the number of repeat entries, and the number of people with banning orders who attempt to enter. Data on underage patrons will not be reliably available. As of 1 July 2017, under the Policy, ID scanners will be mandatory after 10 pm in late trading venues (trading after midnight) located in Safe Night Precincts. While all venues will be required to have ID scanners in place, the units have been popular in licensed venues across Australia for over 5 years, and a wide range of venues around the country have been using them since around 2007 [40, 41]. The researchers have been in contact with major ID scanner supplies (e.g., Scantek) and negotiated access to de-identified unit records. These records will allow us to retrospectively investigate trends in the number of people attending specific licensed venues which used ID scanners across Queensland. While not a perfect measure for prevalence, the data will provide insight into any substantial changes in people attending nightlife licensed venues.

##### Queensland school alcohol- and drug-related violence program audit

One element of the community education component of the Policy involves compulsory education in Queensland schools about alcohol- and drug-related violence. There is no set curriculum; resources are provided via a website developed by the Queensland Department of Education and schools are able to choose which components, if any, they will utilise. To assess this component of the Policy we will conduct an audit of Queensland secondary schools to determine what, if any, violence-specific and alcohol/drug education campaigns have been employed. All secondary schools in Queensland, including private and public schools, will be contacted by telephone or email and asked to outline the education programs currently used in the school, the process through which the education is delivered and the usefulness of the website.

## Analysi**s**

### Administrative data analysis

In order to measure changes over time in secondary data outcome measures, we will use time series analysis approaches including autoregressive integrated moving-average (ARIMA) models [42]. Such models allow for identification and adjustment for underlying trends in the data, seasonal variation, and the serial autocorrelation between observations obtained at different time points. The time series design is a commonly employed approach to the evaluation of policies implemented in entire jurisdictions where suitable control sites are lacking [43]. Hypotheses can be tested regarding the likely rapidity of onset and duration of change by specifying tests for intercept and slope. We will use monthly data over the 5-year period pre-intervention and 3 years post-intervention to model for outcomes of police recorded assaults, ED presentations, and ambulance attendance. This evaluation will use both pre-post within-site comparisons and intra- and inter-state comparisons to gain a greater level of insight into the nature of change occurring across nightlife in Queensland. Due to the low number of expected records, courts and coroners data will be analysed descriptively.

#### Interviews and observations analysis

Data from patron interviews will be analysed using descriptive statistics and regression models: linear, logistic, or Poisson, as appropriate for the items examined [28]. Key informant interviews will be analysed using thematic analysis (identifying common themes in textual data) (e.g. [2, 31, 32, 44]). Observer-rated fields recorded in venue observations will be reported as descriptive frequencies, means and percentages.

#### ID scanner and foot traffic data

ID scanner and foot traffic count data will be analysed using time series models. This data will also be used to compute population density estimates and alcohol-related harms incidence rates.

## Discussion

The Queensland Alcohol-related violence in the Night Time Economy Monitoring (QUANTEM) project extends on previous evaluation frameworks by incorporating a wide variety of data sources to examine the impact of alcohol policy implementation. The study goes beyond the assessment of administrative data and key indicators of alcohol related harms, such as assaults and alcohol-related injuries, to consider the broader financial and cultural effects of policy change. Important elements of the approach include venue observations, and patron and key informant interviews that serve to contextualise quantitative findings and help to better understand the moderating influence of previously ‘invisible’ factors (e.g. population density in night time spaces; physical aspects of night time spaces and the mix of alcohol/non-alcohol businesses) on the relationship between alcohol regulations and alcohol-related harms. This framework brings together data to address questions of concern to a number of stakeholder groups, including liquor licensees, the music industry, and community safety advocates, regarding the effect of the Policy on changes in alcohol-related harms, cultural events/live music, patron, and industry experiences.

By drawing on previous studies conducted in Victoria, Australian Capital Territory, Western Australia, Tasmania, and New South Wales [45–47], this research provides an opportunity to examine the way in which context moderates the effect of policy on alcohol-related harms and will provide insight into how different alcohol policies might have different impacts in different jurisdictions. This study has the potential to inform policy development and will have practical implications for the policing of night time entertainment precincts. In particular, the integration of foot traffic counts and police tasking data with outcome data (e.g., assault offences recorded by QPS (single record per incident); alcohol-related injury) will provide the best estimate to date of incidence rates in the night time economy.

### Limitations

All site comparisons come with limitations; no single site is a perfect comparison for another. However, previous studies have shown the utility of using multiple comparisons to document trends [2, 3, 14, 48–52]. In the Newcastle example, comparisons with nearby Hamilton, which had different characteristics, but similar legislative conditions allowed a part of the picture to be described [49]. Further comparisons with other cities of similar sizes ad demographics, but with no restrictions, will allow a different part of the picture to be described [28, 53]. Further, this study does not look at potential impacts on longer term outcomes (e.g., liver cirrhosis) that may arise due to a reduction in overall alcohol use across the population.

### Conclusions

This study will provide a comprehensive multifaceted evaluation of trends associated with changes in availability of alcohol and enforcement in the night-time economy within Australia, extending previous work to include a range of new methodologies and technologies. The breadth of the evaluation across an entire state, and using novel data collection methods as well as more traditional harms, will inform potential responses to intoxication, harm, and offending in the night-time economy.

## Additional file

Additional file 1: Images of maps. The maps are of the Safe Night Precincts in Queensland that are referred to within the paper. (PDF 8544 kb)

## Abbreviations

ABR: Australian Business Register
ABS: Australian Business Survey
ARIMA: Autoregressive integrated moving-average
BAC: Blood alcohol concentration
CBD: Central business district
CPTED: Crime Prevention Through Environmental Design
EARF: Electronic Ambulance Report Form
ED: Emergency department
HAH: High-alcohol hours
NAPEDC: Non-Admitted Patient Emergency Department Care
NSW: New South Wales
OLGR: Office of Liquor and Gaming Regulation
QACIR: Queensland Ambulance Case Information Reporting
QHAPDC: Queensland Hospital Admitted Patient Data Collection
Qld: Queensland
QPRIME: Queensland Police Records and Information Management Exchange
QPS: Queensland police service
QUANTEM: Queensland Alcohol-related violence in the Night Time Economy Monitoring
SA: South Australia
SNP: Safe Night Precinct
Vic: Victoria
WA: Western Australia
