# Supplementary material for: Queensland Alcohol-related violence and Night Time Economy Monitoring project (QUANTEM): a study protocol
Source: BMC Public Health. 2017 Oct 5;17:789. doi: 10.1186/s12889-017-4811-9 (PMC5629755; doi:10.1186/s12889-017-4811-9)
Supplement: Additional file 1: — Images of maps. The maps are of the Safe Night Precincts in Queensland that are referred to within the paper. (PDF 8544 kb) [file 12889_2017_4811_MOESM1_ESM.pdf]

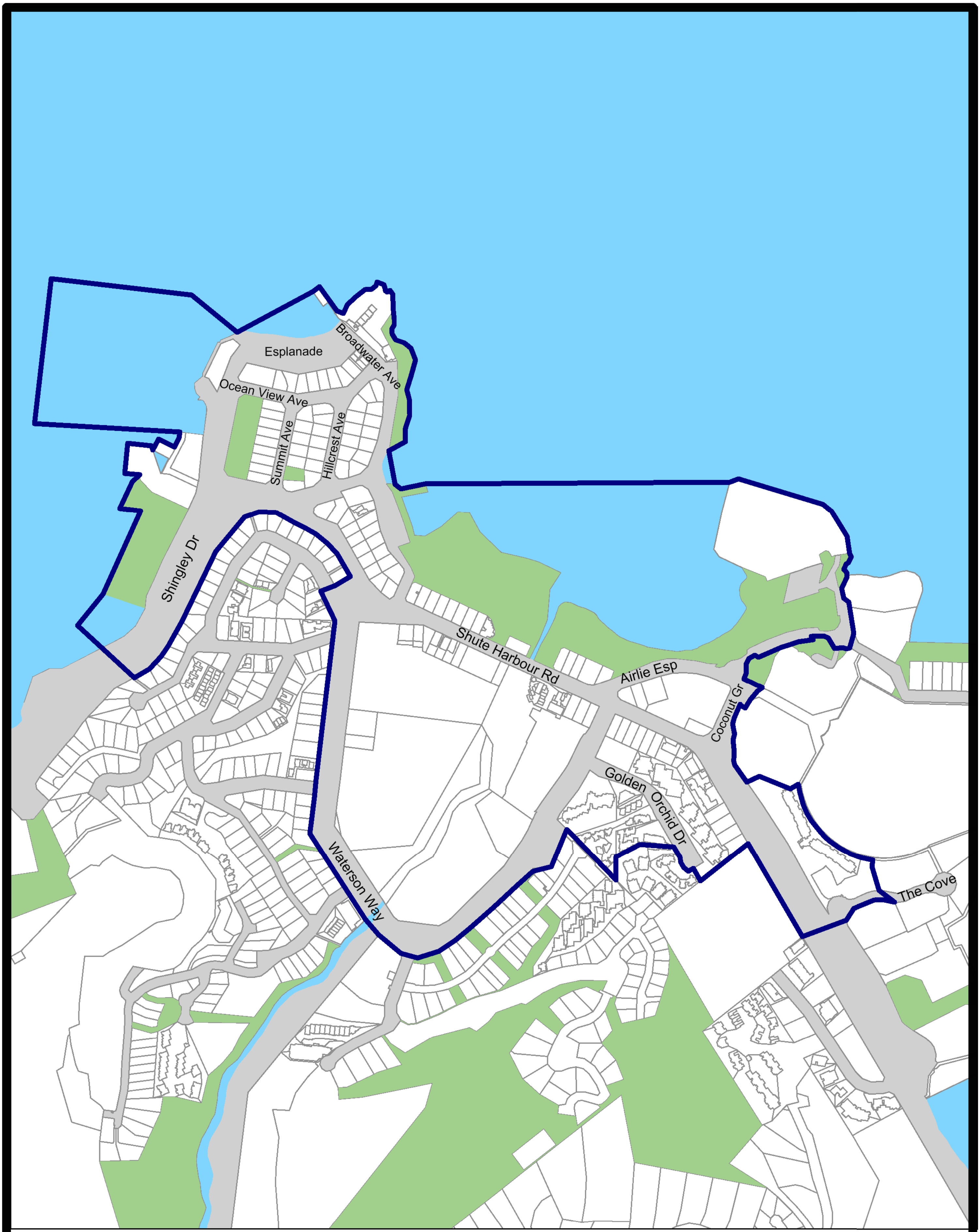

SCALE:

0 150 300 450

Meters

Scale: 1:8,290

N

LEGEND:

- Boundary of safe night precinct
- Waterway
- Parks and reserves
- Property boundaries
- Road casements

AIRLIE BEACH CBD

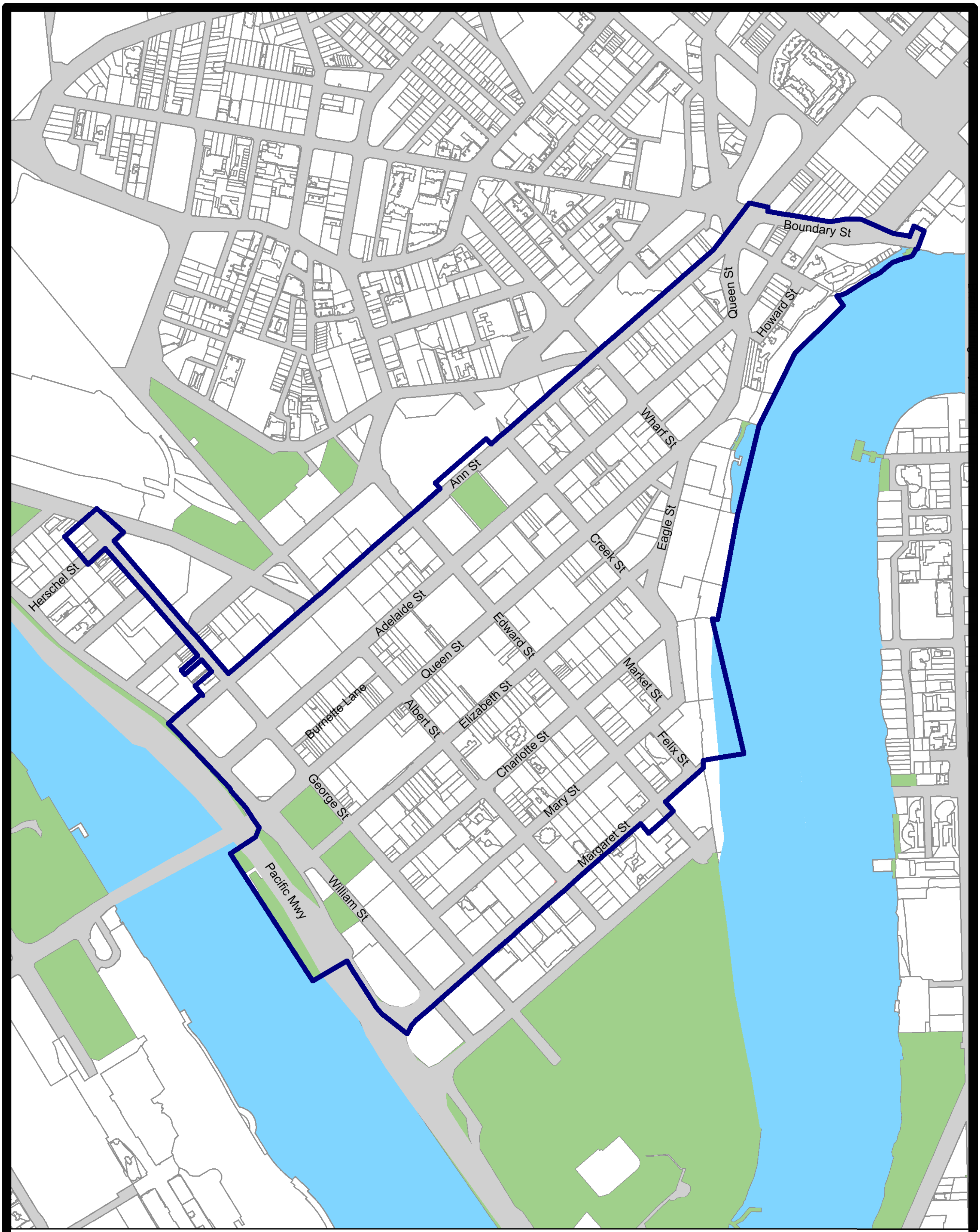

SCALE:

0 150 300 450

Meters

Scale: 1:8,870

N

LEGEND:

- Boundary of safe night precinct
- Waterway
- Parks and reserves
- Property boundaries
- Road casements

BRISBANE CBD

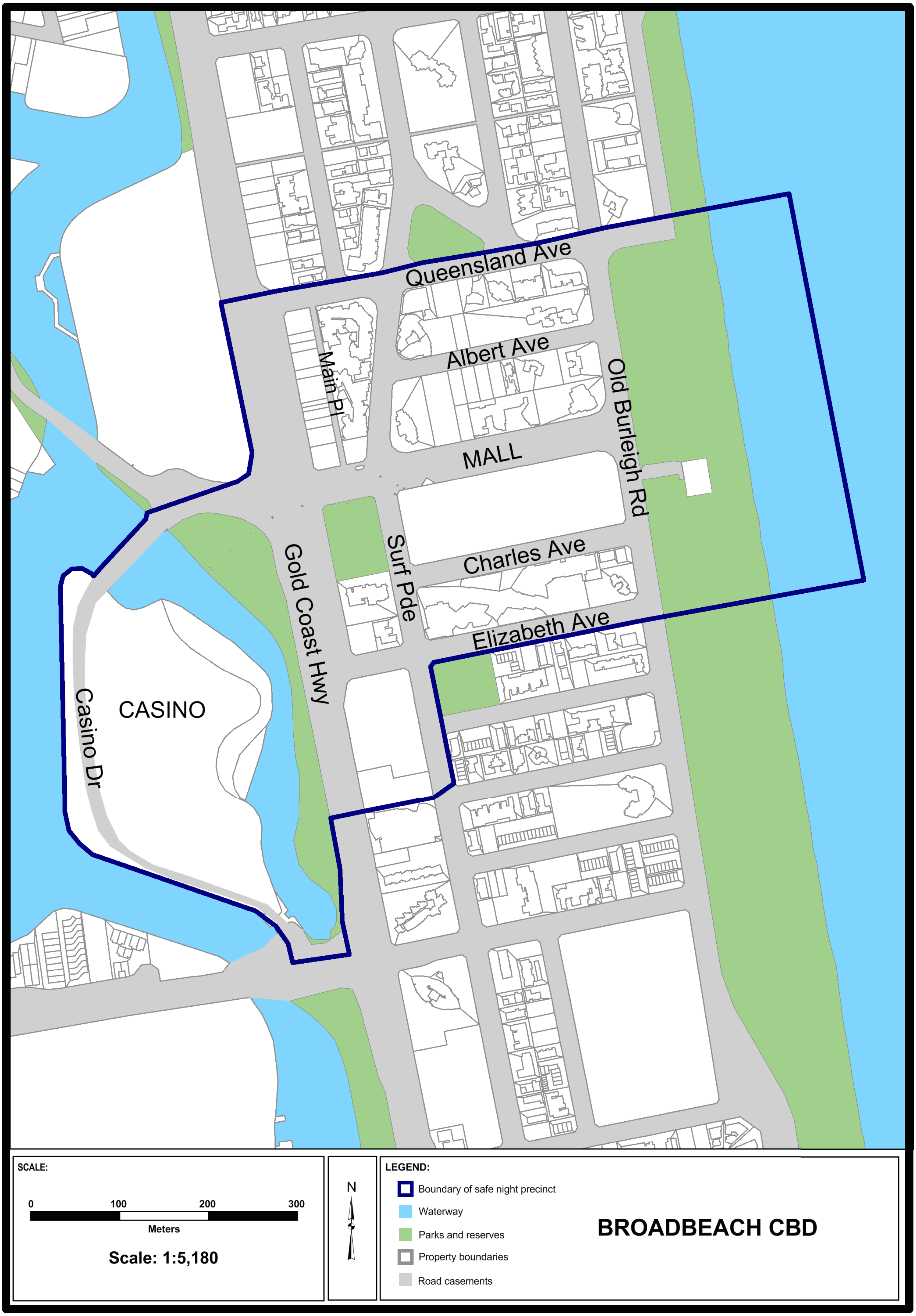

BROADBEACH CBD

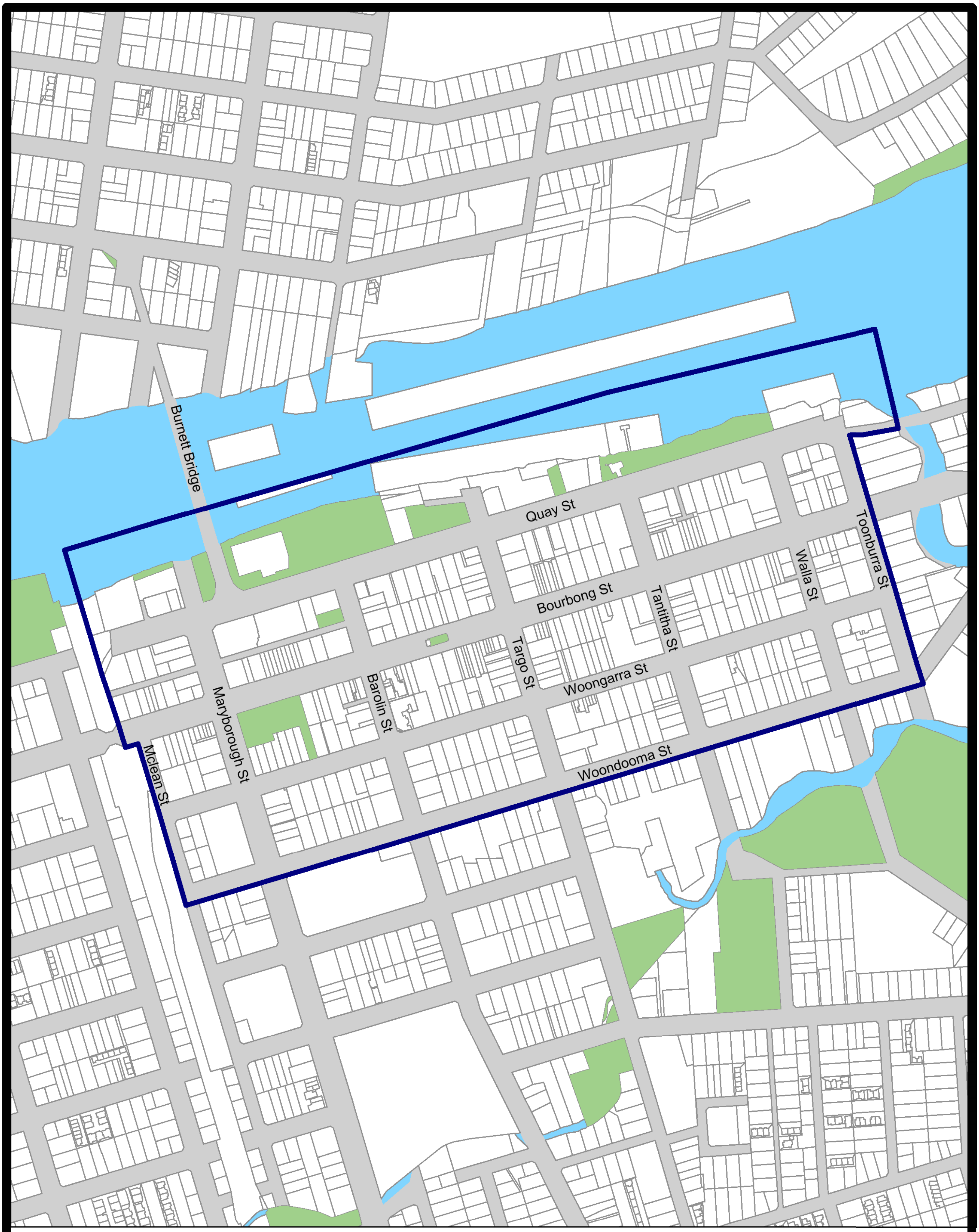

SCALE:

0 150 300 450

Meters

Scale: 1:7,670

N

LEGEND:

- Boundary of safe night precinct
- Waterway
- Parks and reserves
- Property boundaries
- Road casements

BUNDABERG CBD

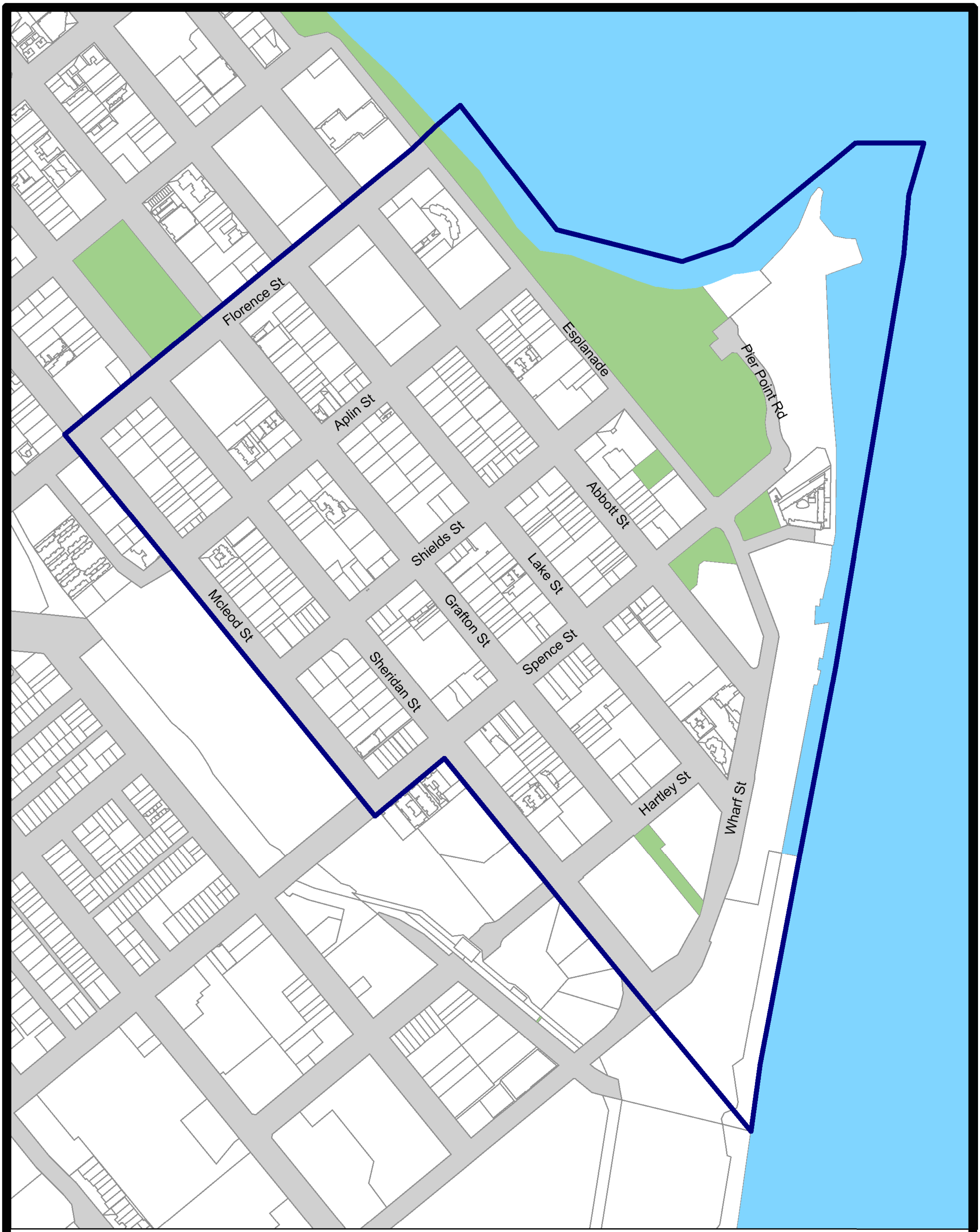

SCALE:

0 100 200 300 400

Meters

Scale: 1:7,710

N

LEGEND:

- Boundary of safe night precinct
- Waterway
- Parks and reserves
- Property boundaries
- Road casements

CAIRNS CBD

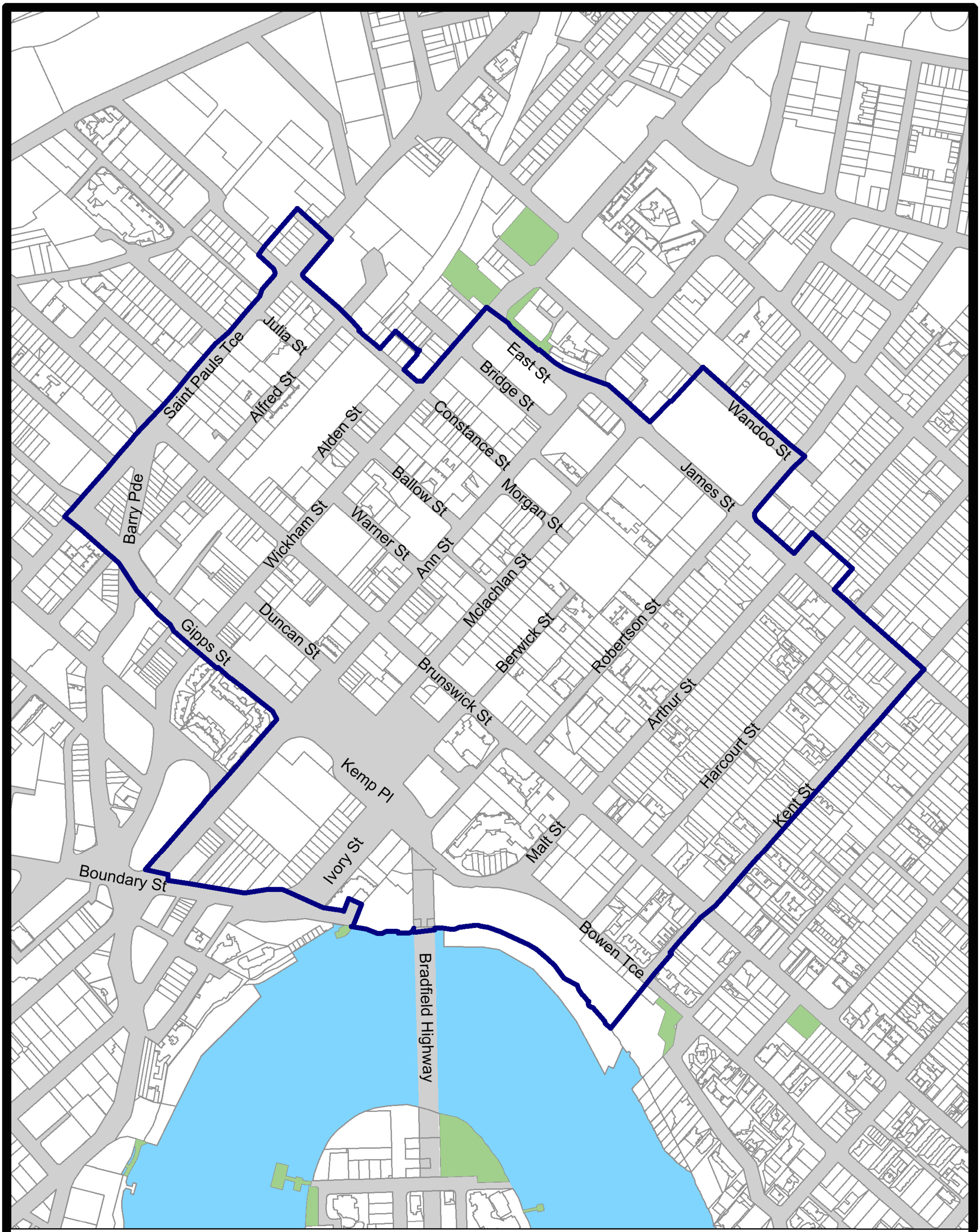

SCALE:

0 100 200 300

Meters

Scale: 1:7,170

N

LEGEND:

- Boundary of safe night precinct
- Waterway
- Parks and reserves
- Property boundaries
- Road casements

FORTITUDE VALLEY

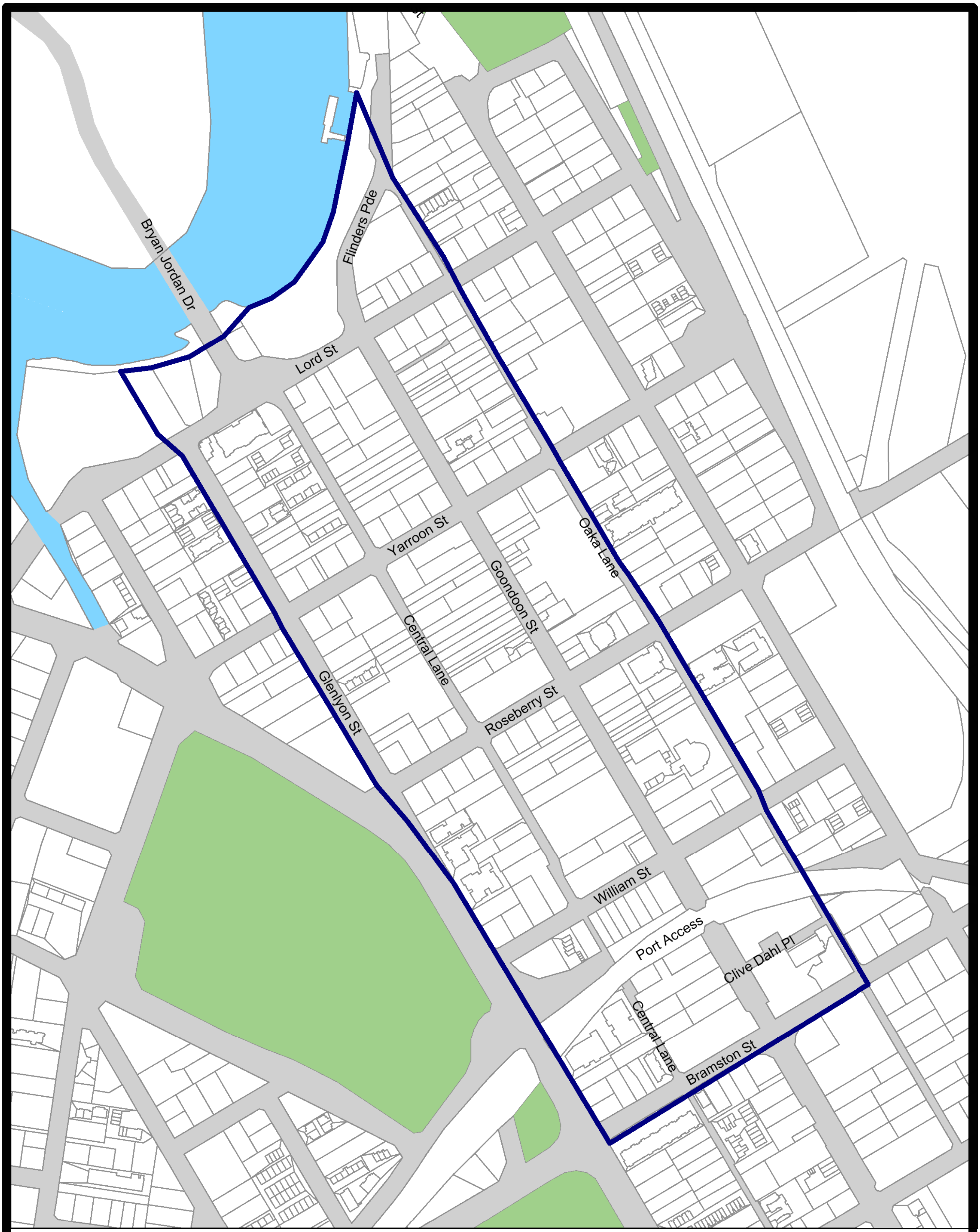

SCALE:

0 100 200 300

Meters

Scale: 1:5,420

N

LEGEND:

- Boundary of safe night precinct
- Waterway
- Parks and reserves
- Property boundaries
- Road casements

GLADSTONE CBD

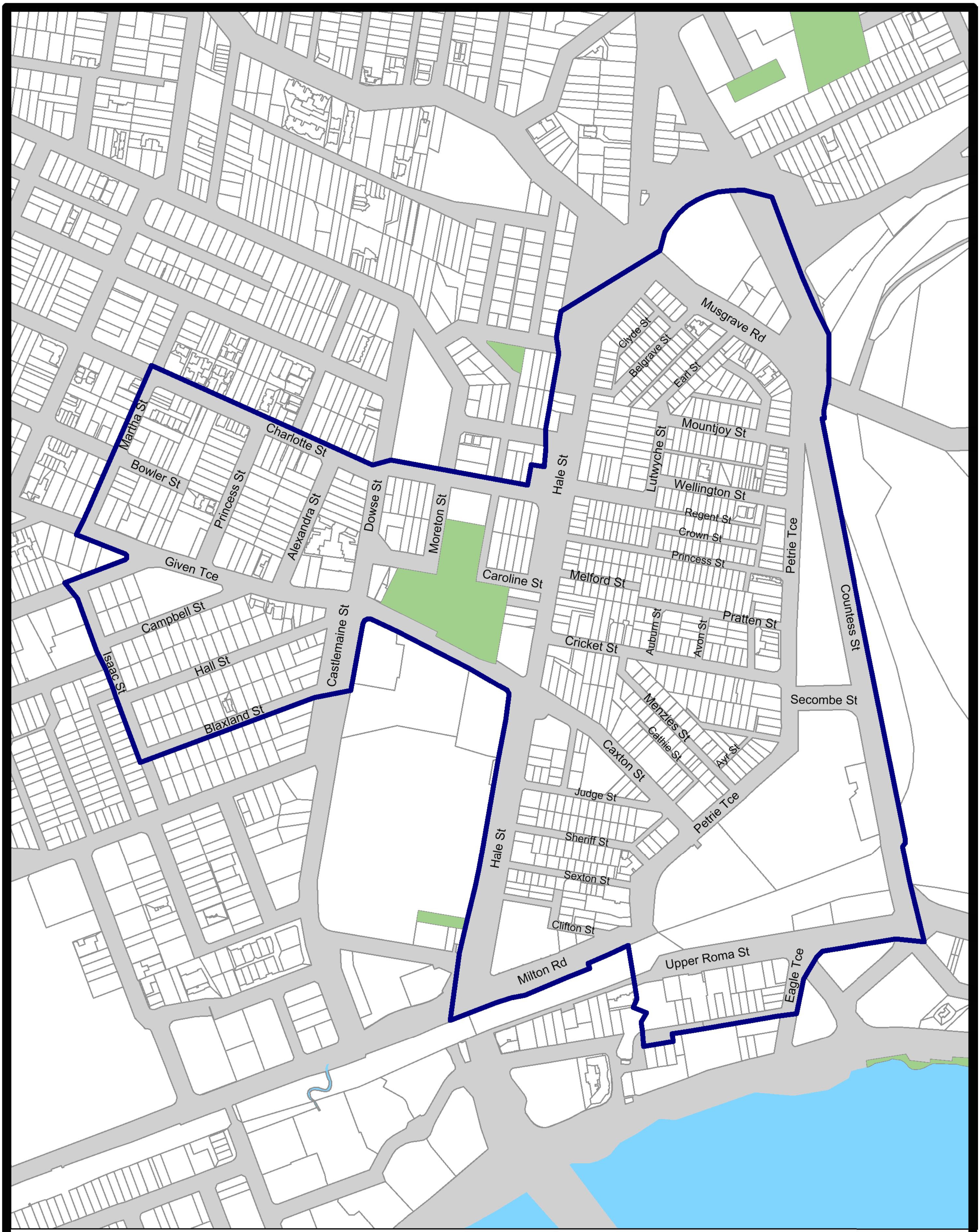

SCALE:

0 100 200 300

Meters

Scale: 1:6,170

N

LEGEND:

- Boundary of safe night precinct
- Waterway
- Parks and reserves
- Property boundaries
- Road casements

INNER WEST BRISBANE

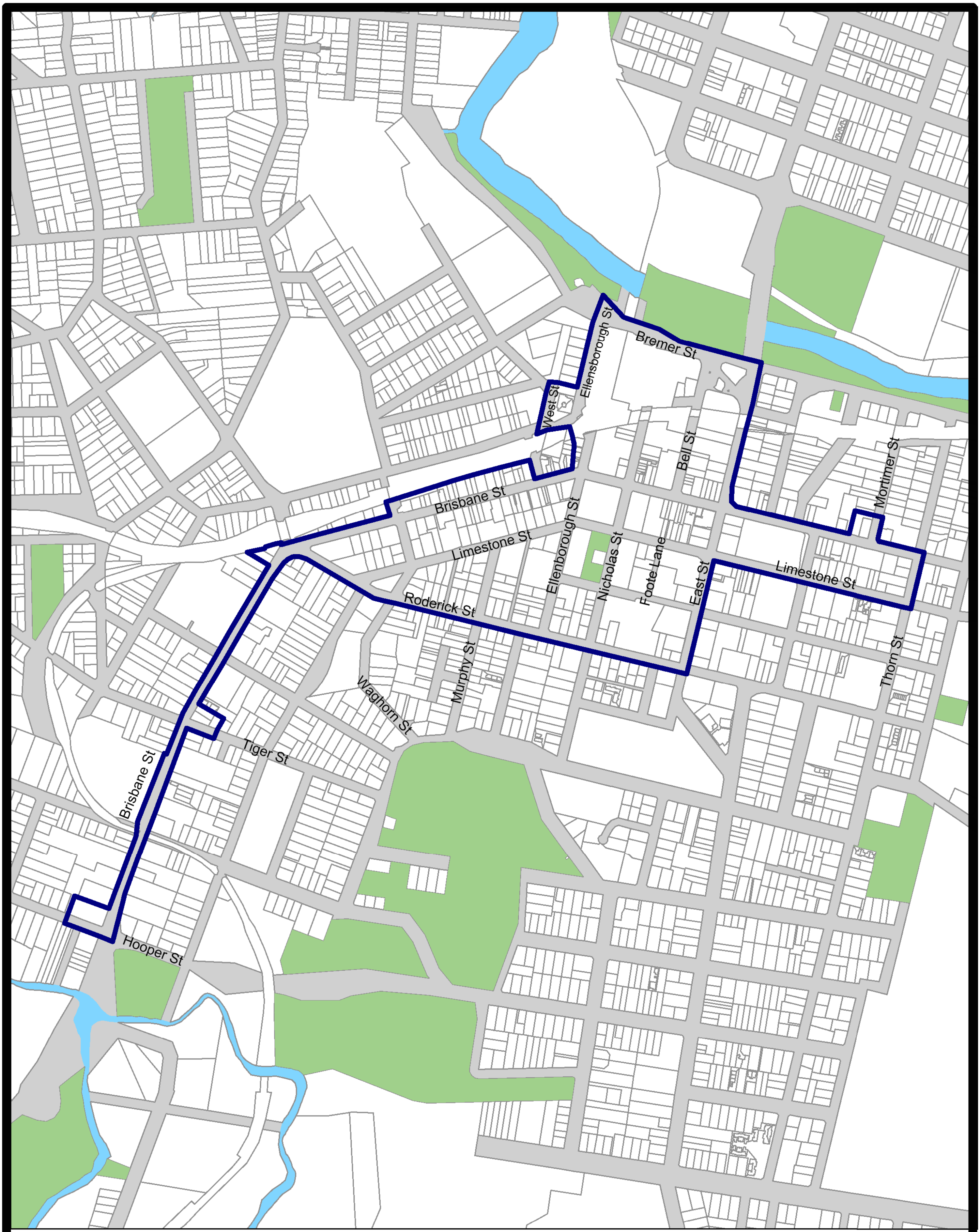

SCALE:

0 200 400 600

Meters

Scale: 1:10,280

N

LEGEND:

- Boundary of safe night precinct
- Waterway
- Parks and reserves
- Property boundaries
- Road casements

IPSWICH CBD

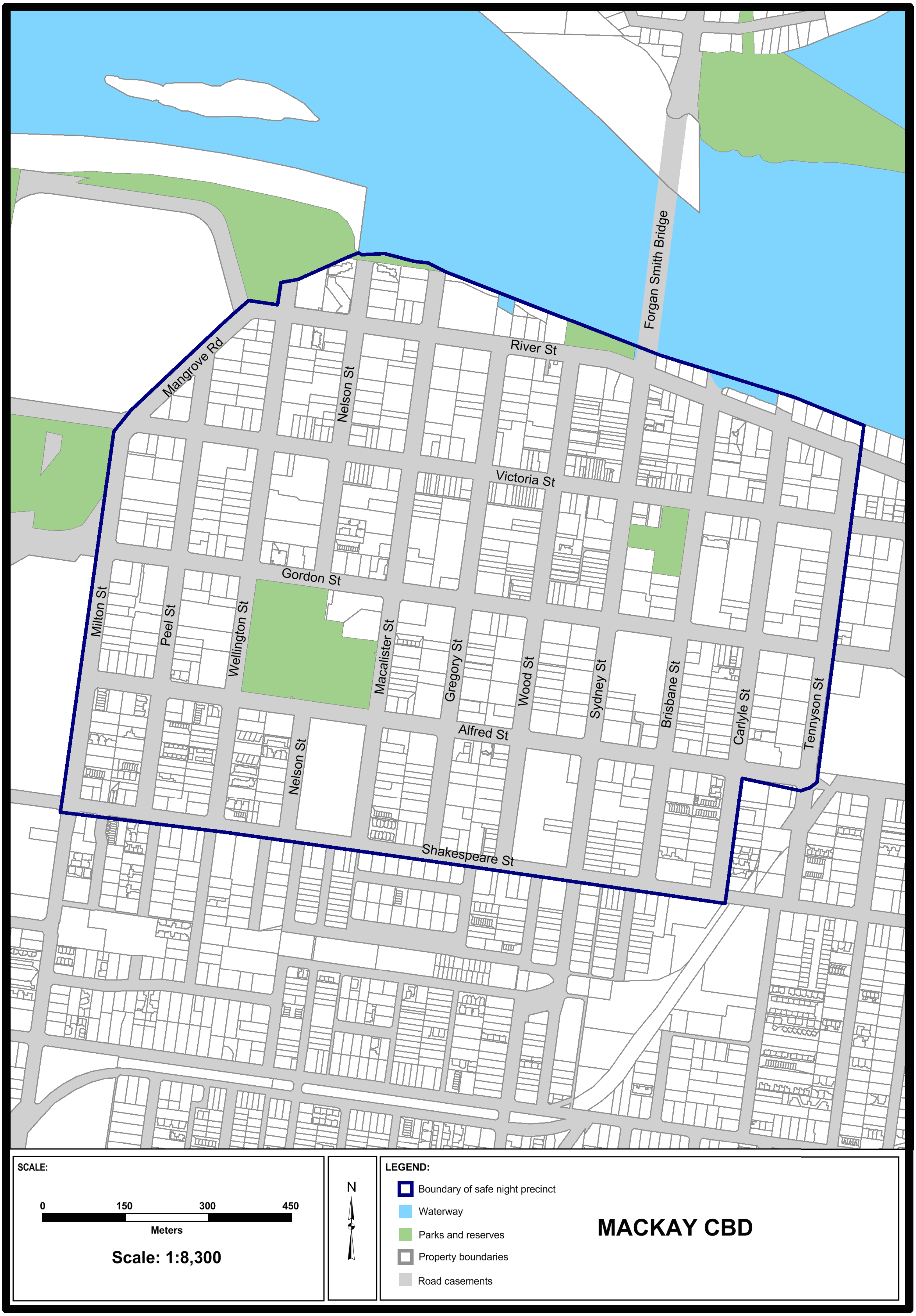
River St
Victoria St
Gordon St
Alfred St
Shakespeare St
Mangrove Rd
Forgan Smith Bridge
Milton St
Peel St
Wellington St
Nelson St
Macalister St
Gregory St
Wood St
Sydney St
Brisbane St
Carlyle St
Tennyson St
SCALE:
0
150
300
450
Meters
Scale: 1:8,300
N
LEGEND:
Boundary of safe night precinct
Waterway
Parks and reserves
Property boundaries
Road casements
MACKAY CBD

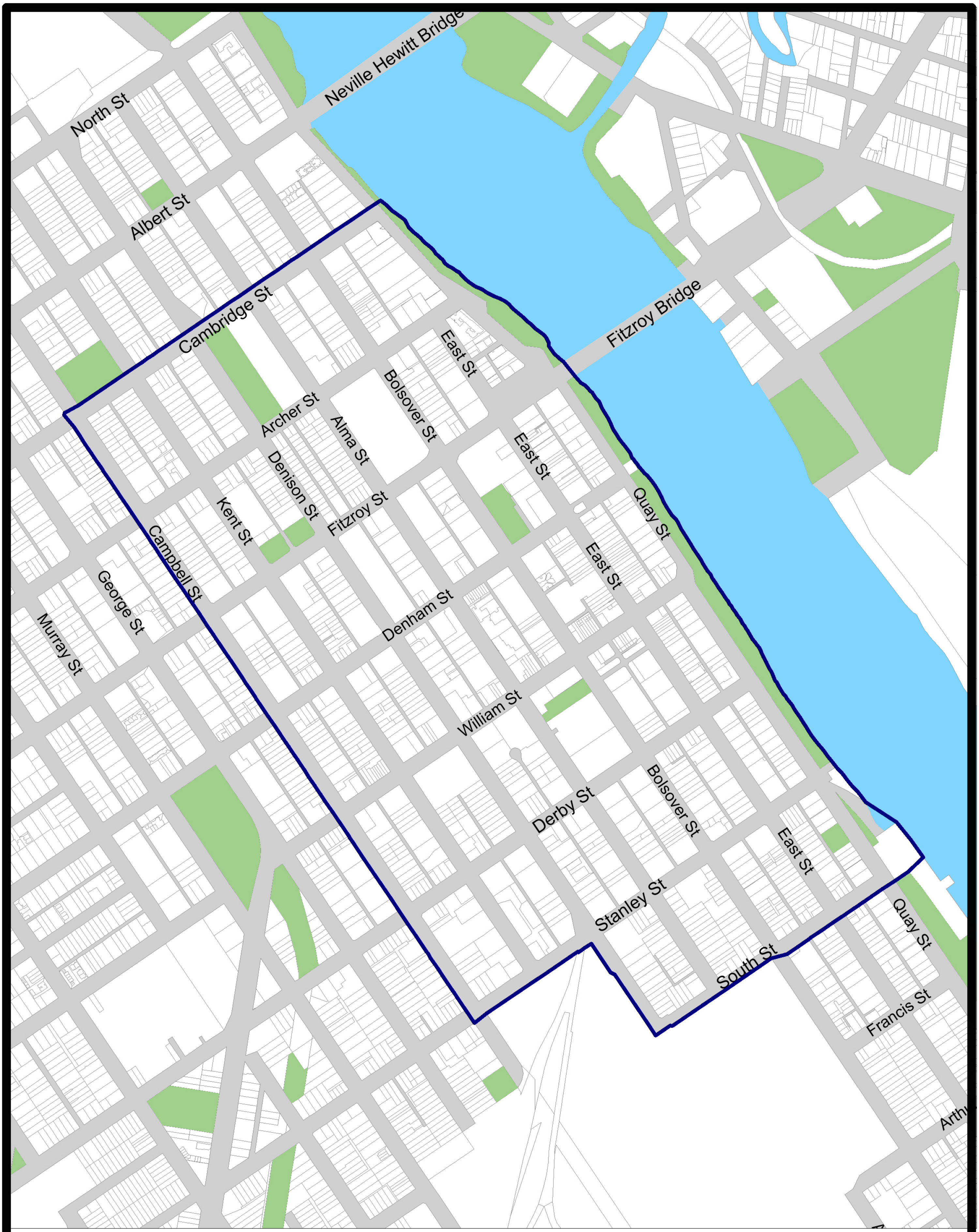

SCALE:

0 250 500

Meters

Scale: 1:10,000

N

LEGEND:

Boundary of safe night precinct

Waterway

Parks and reserves

Property boundaries

Road casements

ROCKHAMPTON CBD

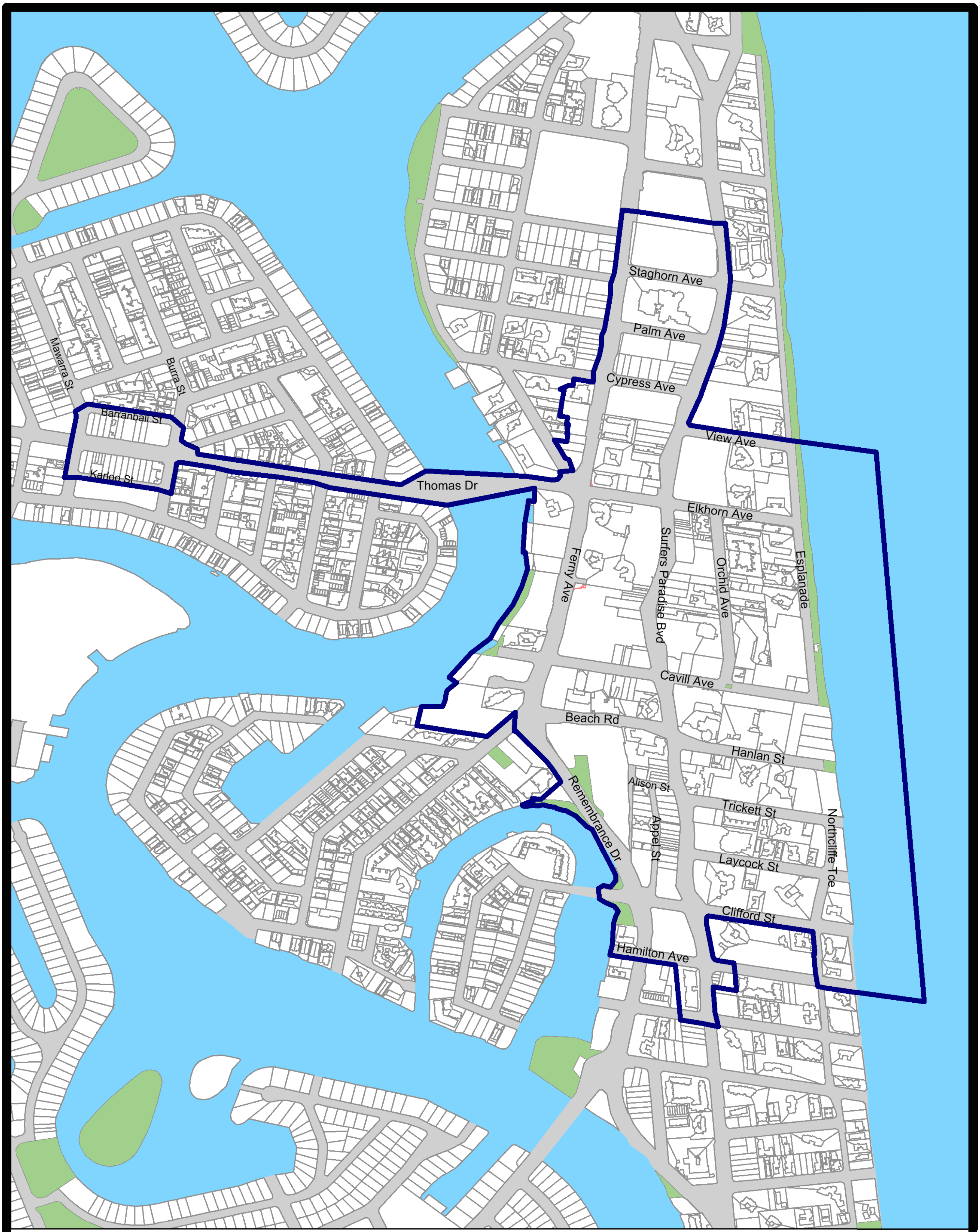

SCALE:

0 150 300 450

Meters

Scale: 1:8,250

N

LEGEND:

- Boundary of safe night precinct
- Waterway
- Parks and reserves
- Property boundaries
- Road casements

SURFERS PARADISE CBD

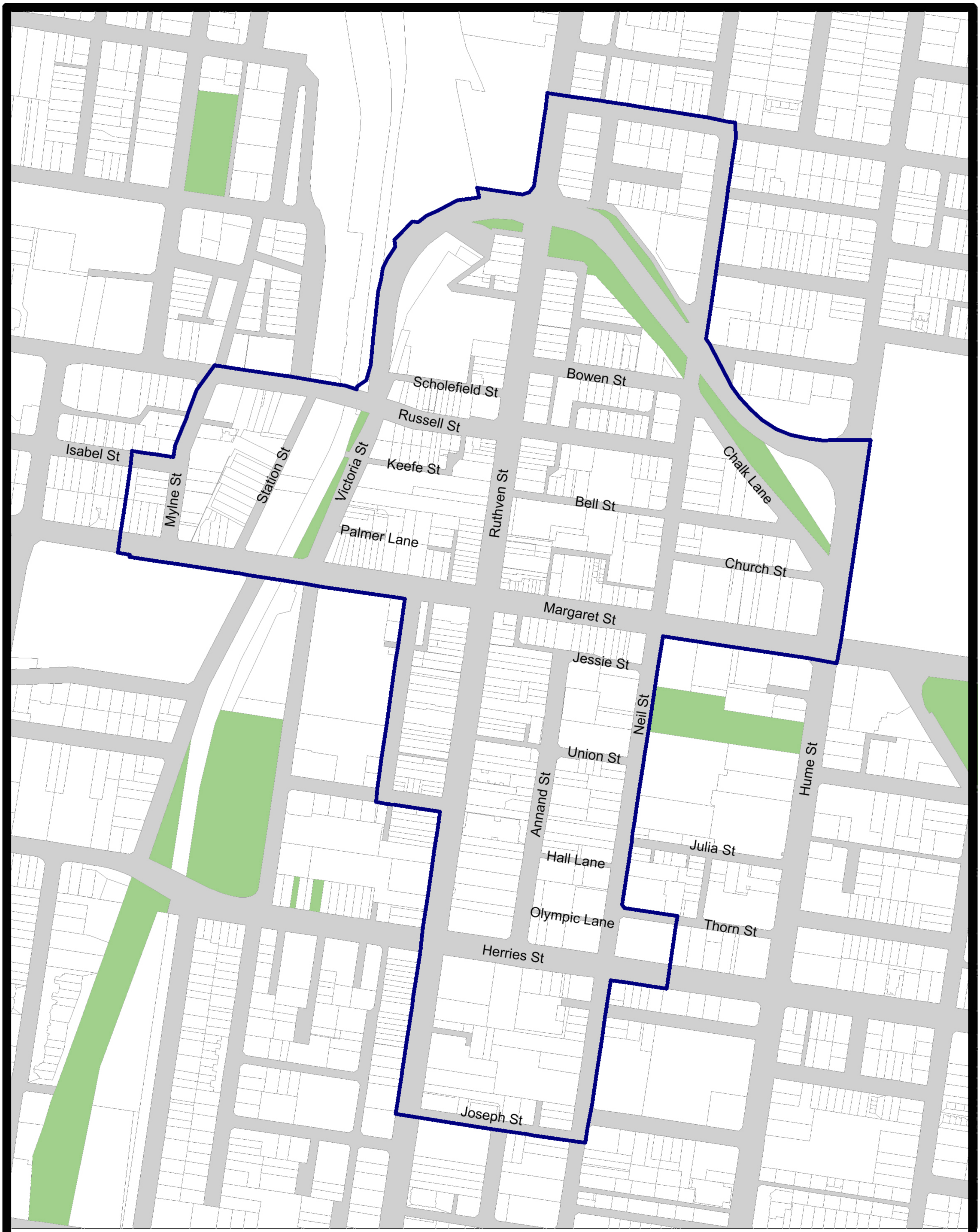

SCALE:

0 100 200 300

Meters

Scale: 1:6,144

N

LEGEND:

- Boundary of safe night precinct
- Waterway
- Parks and reserves
- Property boundaries
- Road casements

TOOWOOMBA CBD

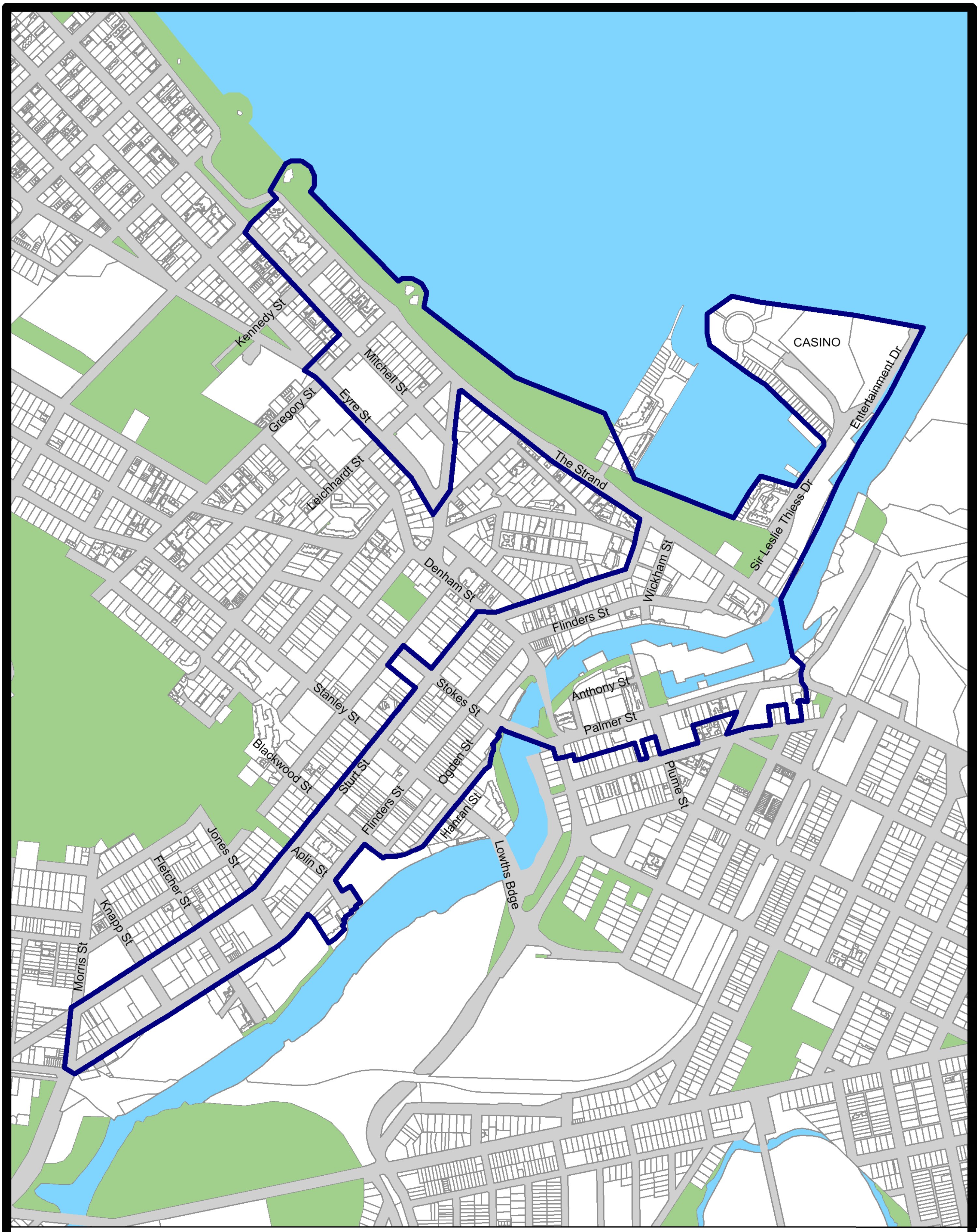

SCALE:

0 250 500 750

Meters

Scale: 1:13,700

N

LEGEND:

- Boundary of safe night precinct
- Waterway
- Parks and reserves
- Property boundaries
- Road casements

TOWNSVILLE CBD

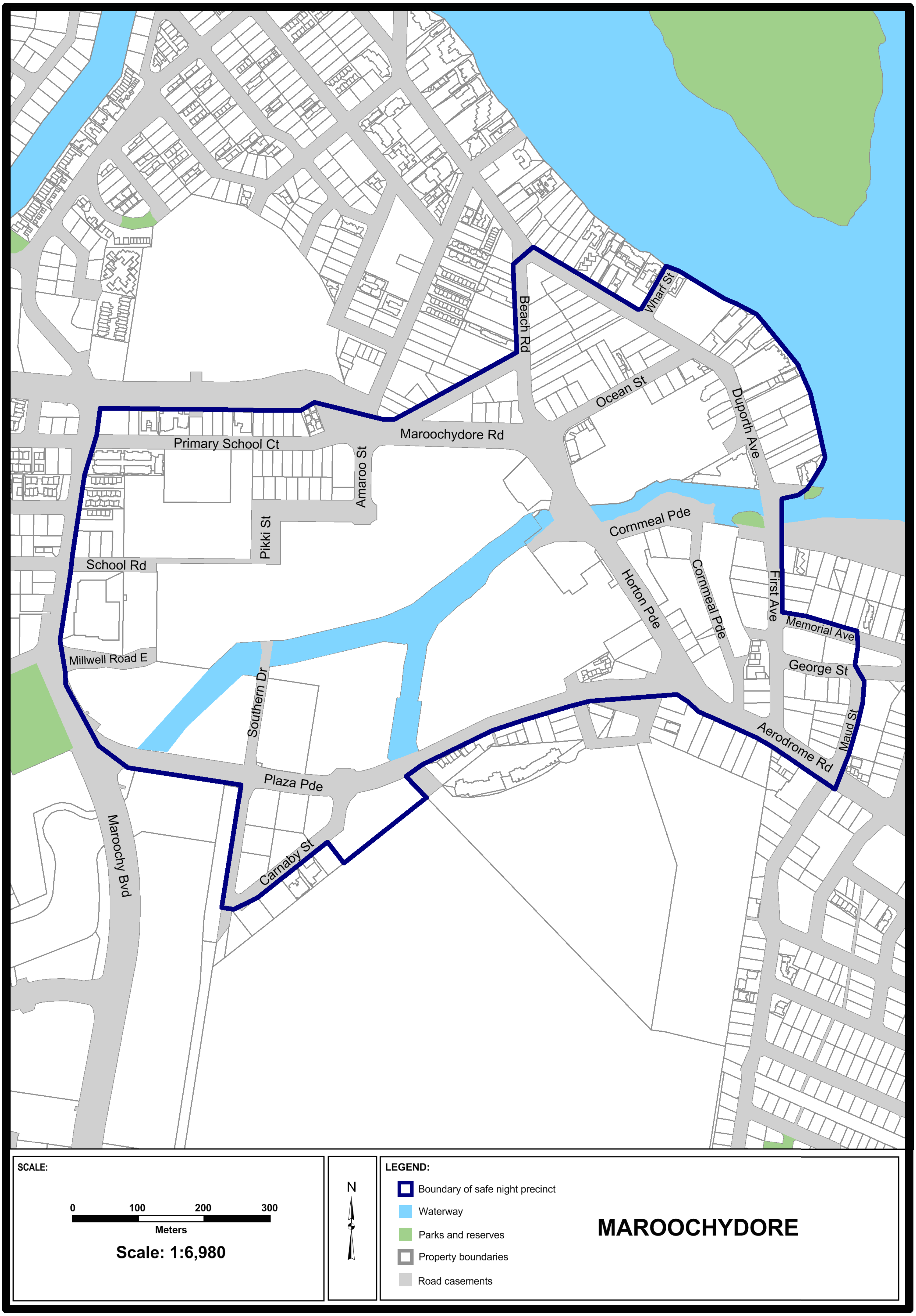
Beach Rd
Wharf St
Ocean St
Duporth Ave
Maroochydore Rd
Primary School Ct
Amaroo St
Pikki St
School Rd
Cornmeal Pde
Horton Pde
Cornmeal Pde
First Ave
Memorial Ave
George St
Maud St
Aerodrome Rd
Millwell Road E
Southern Dr
Plaza Pde
Carnaby St
Maroochy Bvd
SCALE:
0 100 200 300
Meters
Scale: 1:6,980
N
LEGEND:
Boundary of safe night precinct
Waterway
Parks and reserves
Property boundaries
Road casements
MAROOCHYDORE

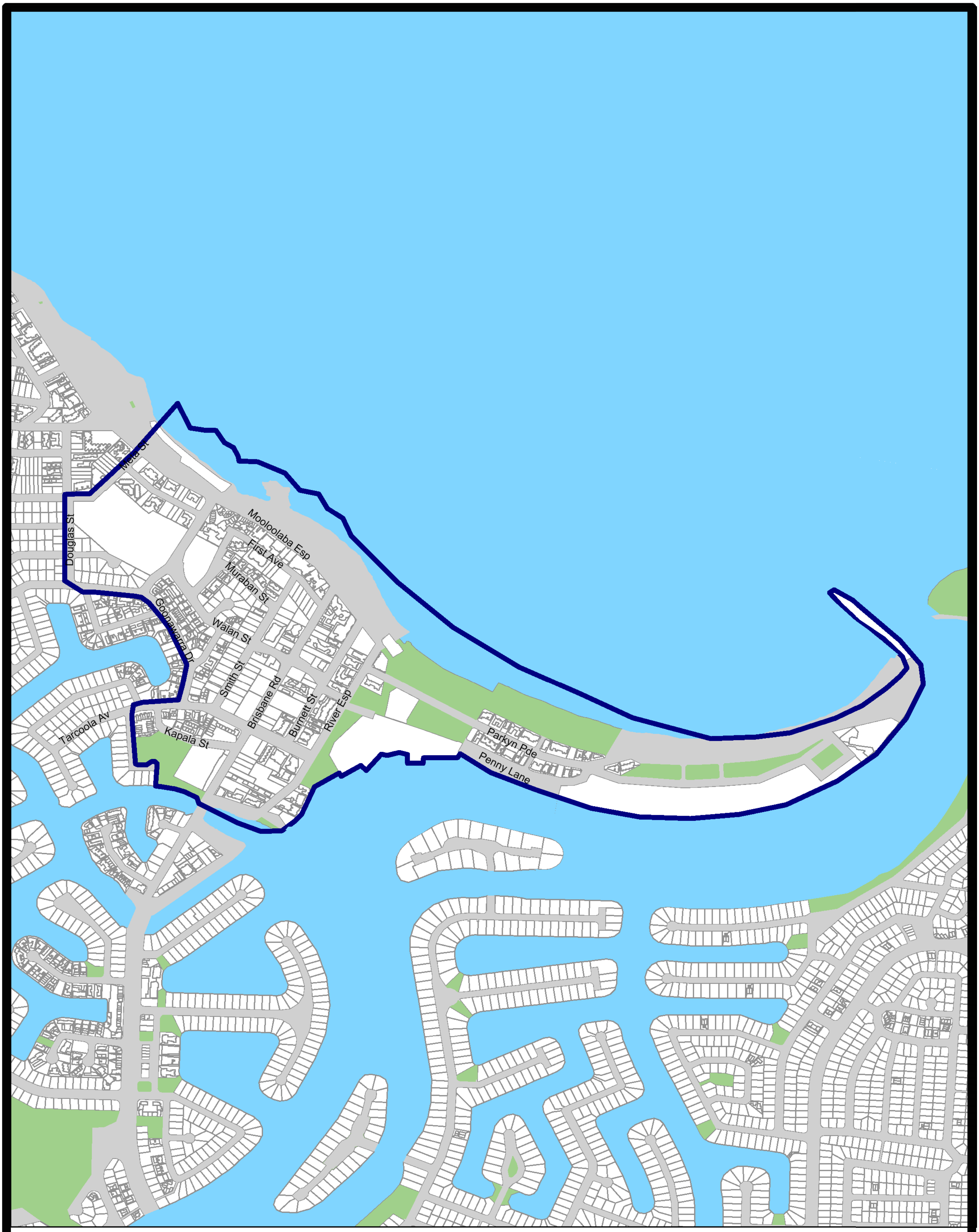

SCALE:

0 200 400 600

Meters

Scale: 1:11,980

N

LEGEND:

- Boundary of safe night precinct
- Waterway
- Parks and reserves
- Property boundaries
- Road casements

MOOLOOLABA

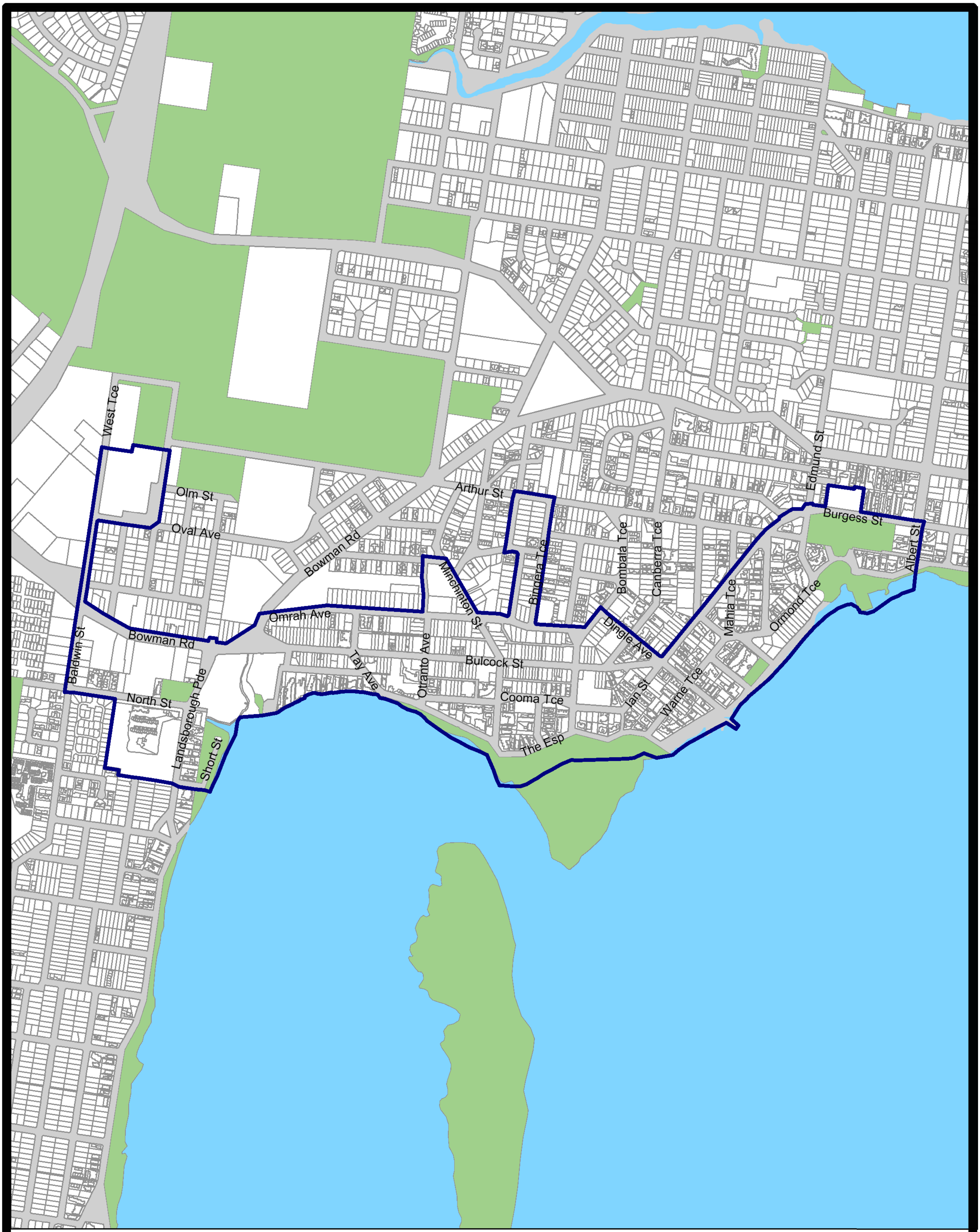

SCALE:

0 250 500 750

Meters

Scale: 1:14,500

N

LEGEND:

- Boundary of safe night precinct
- Waterway
- Parks and reserves
- Property boundaries
- Road casements

CALOUNDRA
